# KY1022, a small molecule destabilizing Ras via targeting the Wnt/β-catenin pathway, inhibits development of metastatic colorectal cancer

**DOI:** 10.18632/oncotarget.13172

**Published:** 2016-11-07

**Authors:** Yong-Hee Cho, Pu-Hyeon Cha, Saluja Kaduwal, Jong-Chan Park, Sang-Kyu Lee, Jeong-Soo Yoon, Wookjin Shin, Hyuntae Kim, Eun Ji Ro, Kyung-Hwa Koo, Ki-Sook Park, Gyoonhee Han, Kang-Yell Choi

**Affiliations:** ^1^ Translational Research Center for Protein Function Control, Yonsei University, Seoul 120-749, Korea; ^2^ Department of Biotechnology, College of Life Science and Biotechnology, Yonsei University, Seoul 120-749, Korea; ^3^ College of Medicine, East-West Medical Research Institute, Kyung Hee University, Seoul 02447, Korea

**Keywords:** Apc mutation, K-Ras mutation, tumor budding, metastatic colorectal cancer, Ras destabilizer

## Abstract

*APC* (80-90%) and *K-Ras* (40-50%) mutations frequently occur in human colorectal cancer (CRC) and these mutations cooperatively accelerate tumorigenesis including metastasis. In addition, both β-catenin and Ras levels are highly increased in CRC, especially in metastatic CRC (mCRC). Therefore, targeting both the Wnt/β-catenin and Ras pathways could be an ideal therapeutic approach for treating mCRC patients. In this study, we characterized the roles of KY1022, a small molecule that destabilizes both β-catenin and Ras via targeting the Wnt/β-catenin pathway, in inhibiting the cellular events, including EMT, an initial process of metastasis, and apoptosis. As shown by *in vitro* and *in vivo* studies using *APC*^*Min*/+^*/K-Ras*^G12D^*LA2* mice, KY1022 effectively suppressed the development of mCRC at an early stage of tumorigenesis. A small molecular approach degrading both β-catenin and Ras via inhibition of the Wnt/β-catenin signaling would be an ideal strategy for treatment of mCRC.

## INTRODUCTION

Colorectal cancer (CRC) is one of the most commonly diagnosed cancers in the world [1]. Although early diagnosis and the development of chemotherapies have increased survival rates of CRC patients, metastasis is still a critical event that renders CRC a lethal disease. In CRC, *Adenomatos polyposis* (*APC*) and *K-Ras* mutations, which have been observed at frequencies as high as 90% and 40-50%, respectively, are major causes of CRC [2–4]. The Wnt/β-catenin and Ras-ERK pathways closely interact during tumorigenesis although the mechanism is poorly understood [5–11]. Stabilization of mutant K-Ras protein (MT-K-Ras) in CRC cells harboring both *APC* and *K-Ras* mutations results in liver metastasis with cancer stem cell activation via strong secondary activation of the Wnt/β-catenin signaling through the MEK-ERK pathway in addition to the initial activation by *APC* loss [9, 10]. Aberrant Wnt/β-catenin and Ras signaling decrease E-cadherin expression, a hallmark of epithelial-mesenchymal transition (EMT), conferring cell motility and invasiveness [12–14], and synergistically increases the invasion capacity of small intestinal tumors in mice harboring the *APC* and *K-Ras* mutations [6]. Therefore, therapies targeting both Wnt/β-catenin and Ras signaling would be an ideal approach for inhibiting CRC metastasis. However, no therapeutic agent targeting the Wnt/β-catenin pathway is available for clinical use.

Recently, selective targeting of oncogenic proteins via degradation has been suggested as an ideal approach for the development of anti-cancer drugs [15]. Therefore, β-catenin and Ras, which are aberrantly stabilized in CRC, could serve as good targets for the development of anti-CRC drugs. Based on our studies, which identified the mechanism of Ras degradation via inhibition of the Wnt/β-catenin pathway [7, 16, 17], we recently identified and characterized small molecules destabilizing both β-catenin and Ras by screening a library of chemicals that inhibit the Wnt/β-catenin pathway [18]. KY1220 and its functionally improved analog KYA1797K specifically bind to the RGS domain of Axin, activate GSK3β via a conformational change enhancing β-catenin complex assembly, and subsequently degrade both β-catenin and Ras via proteasomal degradation [18]. KYA1797K suppressed the formation and growth of CRCs harboring *APC* and *K-Ras* mutations as shown by both *in vitro* and *in vivo* studies [18]. However, the effect of these small molecules destabilizing both β-catenin and Ras on metastasis is unknown.

In this study, we identified that KY1022 as the most effective anti-metastatic drug suppressing the motility and growth of CRC cells among the small molecules that efficiently degrade both β-catenin and Ras via targeting the Wnt/β-catenin pathway [18]. Destabilization of β-catenin and Ras by KY1022 was achieved by a different mode of action with KY1797K. KY1022 significantly inhibited EMT in CRC cells harboring *APC* and *K-Ras* mutations and *APC*^*Min*/+^*/K-Ras*^*G12D*^*LA2* hybrid mice. Our study suggests that destabilization of β-catenin and Ras via targeting Wnt/β-catenin pathway could be an effective approach for treating mCRC patients harboring *APC* and *K-Ras* mutation.

## RESULTS

### Both β-catenin and Ras protein levels are highly increased in tumor budding regions of human adenocarcinoma, and KY1022, a small molecule that degrades both β-catenin and Ras via targeting the Wnt/β-catenin signaling, is identified as an inhibitor of migration of LoVo CRC cells

**Figure 1:**
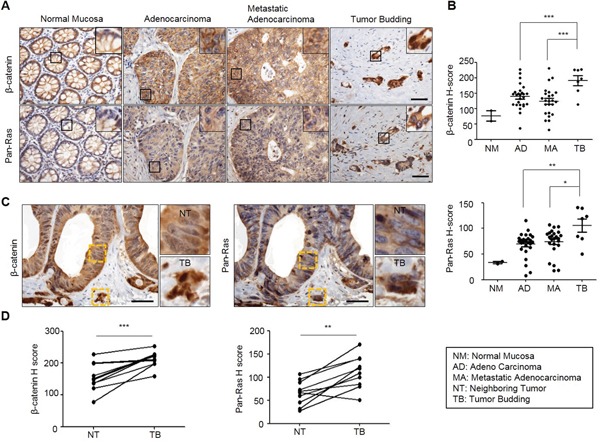
Both β-catenin and Ras levels are increased in adenocarcinoma, metastatic adenocarcinoma and tumor budding in colon cancer TMA specimens of normal mucosa, adenocarcinoma, metastatic adenocarcinoma, or tumor buddings were subjected to the IHC analyses by using β-catenin or Ras antibody followed by 3, 3'-diaminobenzidine (DAB) staining. **A.** Representative image of IHC analyses for β-catenin and Ras in normal tissues and at different stages of colorectal tumorigenesis. Boxes indicate the enlarged areas. Scale bar= 50μm. **B., D.** Quantitative analyses of positive signal were performed by comparing the H-Scores of staining for β-catenin and Ras in TMA samples. Normal Mucosa (n=2), Adenocarcinoma (n=26), Metastatic adenocarcinoma (n=24), Tumor budding (n=7). **C.** Representative images of IHC analyses for β-catenin and Ras in tumor budding compared with a neighboring adenocarcinoma in colon cancer TMA samples. The yellow boxes represent enlarged in the right panel. Scale bar= 50μm. **D.** Quantitative analysis for β-catenin and Ras was performed by comparing tumor budding with neighboring adenocarcinoma based on H-Score (n=10). Two-sided Student t test was used to determine statistical significance using GraphPad Prism5 Software. Error bars represent 95% confidence intervals.

**Figure 2:**
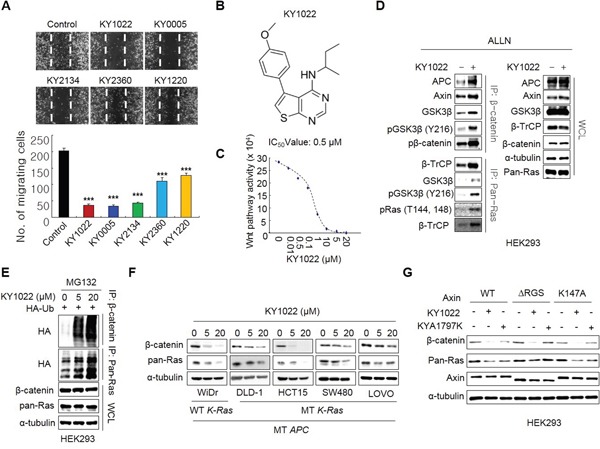
Identification of KY1022, a small molecule that degrades both β-catenin and Ras via targeting Wnt/β-catenin signaling, as an inhibitor of cell motility in LoVo CRC cells **A.** The inhibition effect of cell motility of each compounds was measured by monitoring cell migration using real-time imaging. LoVo cells were grown until confluent, scratched, and treated with each compound. After 18 hours, images were captured from the video file (upper panel) and quantified (bottom panel). **B.** Structure of KY1022. **C.** An inhibition curve for determining the IC_50_ of KY1022 for Topflash activity. HEK293 reporter cells were grown and incubated with various concentrations of KY1022 in Wnt3a-conditioned media (Wnt3a-CM) for 24 hours before harvesting for the measurement of TOPflash activity. **D.** Immunoblots of immunoprecipitation (IP) with β-catenin or pan-Ras. HEK293 cells were treated with ALLN with or without KY1022 (20 μM) for 12 hours, and whole cell lystates (WCLs) were immunoprecipitated with β-catenin or pan-Ras antibody. **E.** Immunoblots of polyubiquitin-dependent proteasomal degradation of β-catenin and pan-Ras. HEK293 cells were transfected with Flag-Ub and then treated with the proteasomal inhibitor MG132 (20 μM) with KY1022 (0, 5 or 20 μM). **F.** Immunoblots of WiDR, DLD-1, HCT15, SW480 or LoVo cells treated with KY1022 (0, 5 or 20 μM). **G.** HEK293 cells were transfected with either pDEST40-axin1-V5 (WT) or the plasmid harboring mutated axin genes (ΔRGS or K147A) with V5 tag. After 24 hours, the cells were treated with KY1022 (20 μM) or KYA1797K (25 μM) for 24 hours. (D-G). Immunoblotting was performed using the indicated antibodies.

Wnt/β-catenin signaling pathway plays critical roles in the formation of metastasis-related tumor budding, which is often observed in colon adenocarcinoma as forms of a single cell or small cluster of cells [19–22]. Interestingly, we observed that β-catenin as well as Ras protein level was increased in tumor buddings compared with adenocarcinoma and metastatic adenocarcinoma regions where these two proteins were stabilized than normal mucosa [7, 18] (Figure 1A and 1B). Moreover, β-catenin and Ras proteins were even more increased in tumor buddings compared with paired neighboring tumors (Figure 1C). Quantitative analyses using tumor buddings (n=10) showed that β-catenin as well as Ras protein was increased in tumor buddings which express strong and uniform nuclear β-catenin [19] (Figure 1D). Since tumor budding is involved in EMT [19, 21, 22], we aimed to investigate the therapeutic effects of the compounds destabilizing β-catenin and Ras on motility of CRC cells. Three compounds (KY1022, KY0005 and KY2134) which significantly inhibit the migration ability of LoVo CRC cells harboring both *APC* and *K-Ras* mutations were identified (Figure 2A). Among these compounds, KY1022 significantly inhibited the cell motility (Figure 2A), reduced the levels of both β-catenin and Ras (Supplementary Figure S1A), and inhibited the growth and transformation of LoVo cells (Supplementary Figure S1B and S1C). The structure of KY1022 consists of a thieno [2, 3-*d*] pyrimidine core, substituted by a secondary butyl amino group at position 4 and a 4-methoxyphenyl group at position 5 (Figure 2B). KY1022 dose-dependently inhibited the TOPflash reporter activity induced by Wnt3a-conditioned medium with an IC_50_ value of 0.5 μM (Figure 2C). KY1022 also efficiently reduced both β-catenin and pan-Ras stabilization by Wnt3a (Supplementary Figure S2A) with increased phosphorylations of β-catenin at S33 and S37 via the activation of GSK3β as evidenced by its phosphorylation at Y216 (Figure 2D). KY1022 also augmented β-catenin binding affinities of APC, Axin, GSK3β, and β-transducin repeats-containing protein (β-TrCP) and also increased GSK3β-mediated phosphorylations of Ras at Thr-144 and -148 (Figure 2D), which are required for degradation of Ras via Wnt/β-catenin signaling [7]. In addition, KY1022 increased the polyubiquitination of both β-catenin and Ras (Figure 2E) without changing their mRNA levels (Supplementary Figure S2B). Both β-catenin and Ras were reduced by KY1022 treatment in various CRC cell lines (WiDR, DLD-1, HCT15, SW480, and LoVo) expressing either wild-type or mutant K-Ras (Figure 2F). KY1022 inhibited the activities of the Ras downstream kinases ERK and Akt in HEK293 cells transiently expressing WT K-Ras or MT K-Ras^G12D^ (Supplementary Figure S3A) as well as in SW480 and LoVo human CRC cell lines harboring *K-Ras^G12V^* and *K-Ras^G13D^*, respectively (Supplementary Figure S3B). However, β-catenin and Ras were not degraded by KY1022 in HCT116 cells harboring non-degradable *β-catenin* mutant (Supplementary Figure S3B) similar to the effect of previously identified small molecule KYA1797K [18]. However, unlike with KYA1797K which functions via binding to RGS domain of axin [18], KY1022 decreased the β-catenin and Ras in HEK293 cells expressing KYA1797K binding mutants of axin (ΔRGS, K147A) (Figure 2G). Moreover, KY1022 increased the amount of APC co-immunoprecipitated with β-catenin (Figure 2D), whereas KYA1797K did not change this affinity [18]. Overall, KY1022 represents a new class of a compound degrading both β-catenin and Ras via targeting Wnt/β-catenin signaling, and inhibits the migration of CRC cells.

### KY1022 induces apoptosis as well as growth inhibition of CRC cells harboring *APC* and *K-Ras* mutations

**Figure 3:**
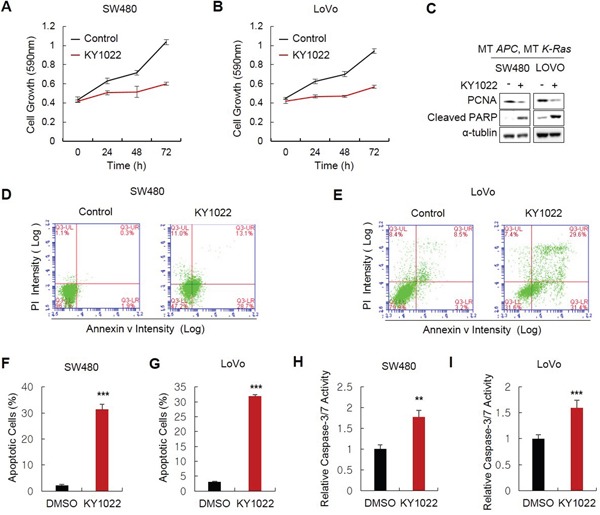
Effects of KY1022 on the growth and apoptosis of CRC cells harboring *APC* and *K-Ras* mutations SW480 and LoVo cells cultured and 20 μM of KY1022 was treated. **A.-B.** MTT assay was performed to determine the effect of KY1022 on cell growth in SW480 (A) or LoVo (B). **C.** Immunoblots of WCLs of SW480 or LoVo cells cultured with or without KY1022 for 72 hours. Immunoblotting was performed using indicated antibodies. **D.-E.** Flow cytometry analyses of SW480 (D) or LoVo (E) cells stained with PI and annexin ν using BD Accuri™ flow cytometer and **F.-G.** Percentage of early apoptotic cells expressing annexin ν and not expressing PI were quantified. **H.-I.** Cysteine-aspartic acid protease-3 (caspase-3) activity were measured. (F-I). Data represent the mean ± s.d. (n=3). *P < 0.05, **P < 0.005, ***P < 0.001 based on the Student's *t* test between control and each sample, respectively.

Wnt/β-catenin and Ras signaling pathways are important in the regulation of proliferation and apoptosis in CRC cells. KY1022 effectively inhibited the growth of SW480 and LoVo CRC cells harboring *APC* and *K-Ras* mutations (Figure 3A and 3B) and reduced level of proliferating cell nuclear antigen (PCNA) (Figure 3C). We tested the effect of KY1022 on the apoptosis of CRC cells due to the involvement of the Wnt/β-catenin and Ras signaling pathways in the regulation of apoptosis [23–25]. As shown by flow cytometry analysis, KY1022 significantly increased the number of early apoptotic cells expressing annexin v, but not propidium iodide (PI) (Figure 3D–3G) and enhanced the Caspase-3/7 activity in SW480 and LoVo CRC cells (Figure 3H and 3I). The apoptosis inducing effect of KY1022 was confirmed by an increase in the level of Poly ADP ribose polymerase (PARP), an apoptosis marker (Figure 3C). Although growth inhibition by KY1022 was significant in SW480 and LoVo cells harboring *APC* and *K-Ras* mutations, we only observed weak growth inhibitory effect of KY1022 on normal colonic fibroblast CCD18-CO and HEK293 cells (Supplementary Figure S4), indicating a specific or at least preferential effect of KY1022 in the suppression of CRC cells specifically harboring *APC* and *K-Ras* mutations.

### KY1022 attenuates tumorigenesis of *APC^Min/+^*/*K-Ras^G12D^LA2* compound mice

**Figure 4:**
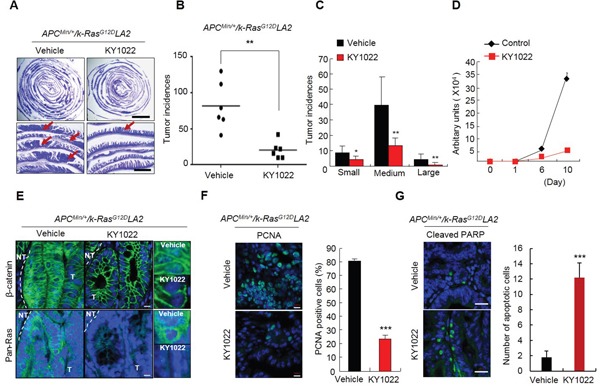
Effects of KY1022 on small intestinal tumorigenesis of *Apc^Min/+^/K-Ras^G12D^*LA2 Five week old C57BL/6J-*Apc*^*Min*/+^*/K-Ras*^*G12D*^*LA2* mice (n=6) were treated with KY1022 (25 mg/kg) or vehicle every day by intraperitoneal (i.p.) injection for 8 weeks. **A.** Swiss roll images of vehicle or KY1022 treated small intestines of C57BL/6J-*Apc*^*Min*/+^*/K-Ras*^*G12D*^*LA2* were stained by H&E and captured by a Nikon SMZ 745T. The scale bar indicates 5 mm (upper panel) 1 mm and (bottom panel). **B.-C.** Tumor incidences (B) and sizes (C) were quantified. Small, medium and large tumors were classified based on diameter as being under 1 mm, between 1 mm and 3 mm, over 3 mm respectively. **D.** Intestinal tumor cells from *Apc*^*Min*/+^*/K-Ras*^*G12D*^*LA2* mice were cultured in N_2_ medium containing ENR condition. One day after seeding, cells were treated with KY1022 (20 μM) for 9 days. Growth curves were measured at 0, 1, 6, 10 day by cell titter assay. Fresh medium with or without KY1022 was changed every 2 days. **E.** The expression of β-catenin (left) and pan-Ras (middle) of the intestinal tumor sections were evaluated by IHC. Nuclei were counterstained with DAPI. Scale bar = 20 μm. Images were captured using an LSM700 (Zeiss) confocal microscope. T, tumor area, NT, non-tumor area. **F.** IHC analysis of PCNA in small intestinal tumors of KY1022 or vehicle injected *Apc*^*Min*/+^*/K-Ras*^*G12D*^*LA2*. Scale bar = 10 μm. Representative images were captured by microscope (LSM700, Carl zeiss) confocal microscope (left). Quantified data (right). **G.** The effect of KY1022 on small intestinal tumors in *Apc*^*Min*/+^*/K-Ras*^*G12D*^*LA2* mice were analyzed by IHC using cleaved PARP antibody. Representative images were captured by confocal microscopy (LSM700, Carl zeiss) (left), and quantified (right). (F-G). Two-sided student *t* test was used to determine statistical significance using GraphPad Prism5 Software. Data represent mean ± s.d. (n=3). *P < 0.05, **P < 0.005, ***P < 0.001. Two sided Student's *t* test was used to determine statistical significance.

We next tested the effect of KY1022 on small intestinal tumorigenesis in the *APC*^*Min*/+^*/K-Ras*^*G12D*^*LA2* compound mouse model [9, 18]. KY1022 effectively inhibited tumor formation by 73.59% with a significant decrease in the sizes of its small intestinal tumors in *APC*^*Min*/+^*/K-Ras*^*G12D*^*LA2* mice (Figure 4A–4C). The growth inhibitory effect of KY1022 has been confirmed using primary tumor cells from small intestinal tumor of *APC*^*Min*/+^*/K-Ras*^*G12D*^*LA2* mice (Figure 4D). Immunohistochemical (IHC) analysis of small intestinal tumors of *APC*^*Min*/+^*/K-Ras*^*G12D*^*LA2* mice showed significant decrease in the levels of nuclear and cytosolic β-catenin as well as those of cytosolic and membraneous Ras by KY1022 treatment (Figure 4E). KY1022 injection decreased the number of proliferating cells in the small intestinal tumors of *APC*^*Min*/+^*/K-Ras*^*G12D*^*LA2* mice by 72% (Figure 4F). In addition, KY1022 injection effectively induced apoptosis in small intestinal tumor cells of *APC*^*Min*/+^*/K-Ras*^*G12D*^*LA2* mice as shown by a 6.8-fold increase in the cells shown PARP cleavage upon KY1022 treatment (Figure 4G). Taken together, KY1022 effectively suppresses the initiation and growth of tumors caused by *APC* and *K-Ras* mutations with significant the increase in apoptosis and a decrease in proliferation.

### KY1022 inhibits the EMT, motility and invasion of D-WT and D-MT cells

**Figure 5:**
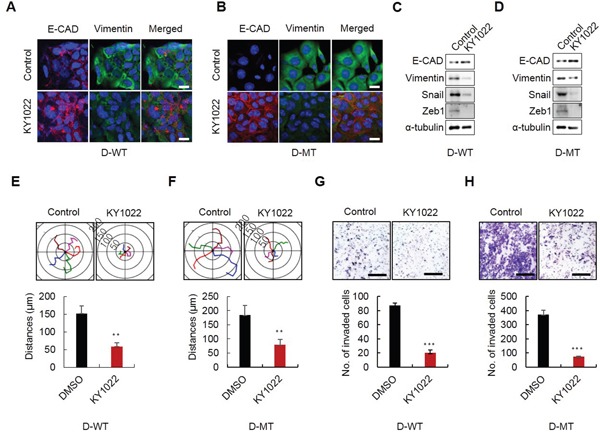
Effects of KY1022 on EMT, motility and invasion in *K-Ras* WT and MT cells D-WT or D-MT cells were grown in RPMI medium containing EGF (20 ng/mL) and treated with KY1022 (20 μM) in D-WT or D-MT cells. **A.-B.** ICC analyses of E-cadherin (red) and vimentin (green) in D-WT (A) and D-MT Cells (B) KY1022 or DMSO treatment and analyzed by a confocal microscope (LSM700, Carl zeiss). Cell nuclei were stained with DAPI. Scale bars = 20 μm. **C.-D.** Immunoblot analyses of WCLs from D-WT (C) or D-MT (D) were performed using the indicated antibodies. **E. -F.** Single-cell migratory behavior was monitored using real-time imaging microscopy (Eclipse *ti*, Nikon) at least five independent times using D-WT (E) and D-MT (F) cells, Collagen coating was used for single cell migration assay. Data represent mean ± s.d. (n=5). **G.-H.** invasion assay was performed using matrigel coated chambers. D-WT (G) and D-MT (H) cells invaded through the matrigel were stained with 0.25% crystal violet. Representative images were captured by Nikon TE2000U (upper), and the total number of invaded cells is quantitated (bottom). (E-H). Data represent mean ± s.d. (n=5). *P < 0.05, **P < 0.005, ***P < 0.001 based on two sided Student's *t* test between control and each sample, respectively.

Tumor budding which has highly enriched β-catenin and Ras protein is associated with EMT (Figure 1). Therefore, we tested the effect of KY1022, which destabilizes these two proteins, on EMT. First, we investigated the effects of destabilization of both β-catenin and Ras by KY1022 on EGF- and bFGF-mediated morphological changes in Madin–Darby canine kidney (MDCK) cells. Upon treatment with both EGF and bFGF, MDCK cells underwent a morphological change to an elongated fibroblastic cell shape which is an indicative feature of EMT. All of these changes were abrogated by treatment with KY1022 and the cell morphology was altered to a cobblestone like appearance (Supplementary Figure S5). Mutations in *K-Ras* have been associated with EMT and insensitiveness to EFGR mAbs such as cetuximab which are often prescribed for treatment of mCRC patients [26–28]. Therefore, we tested whether KY1022 suppresses the metastatic phenotypes in the isogenic DLD-1 harboring wild-type (WT) and mutant (MT) *K-Ras,* D-WT and D-MT cells, respectively [29]. KY1022 significantly inhibited the EMT as evidenced by an increase in E-cadherin and a decrease in vimentin in both D-WT and D-MT cells, respectively (Figure 5A and 5B). The inhibitory effect of KY1022 on EMT in D-WT and D-MT cells was confirmed by immunoblotting analyses (Figure 5C and 5D). Furthermore, KY1022 effectively suppressed the motility and invasion capacities in both D-WT and D-MT cells (Figure 5E–5H). Overall, KY1022 significantly suppressed the EMT and consequently the cell motility and invasion capacities regardless of *K-Ras* mutation status.

### KY1022 inhibits actin rearrangement and suppresses the motility and invasion abilities of CRC cells harboring both *APC* and *K-Ras* mutations

**Figure 6:**
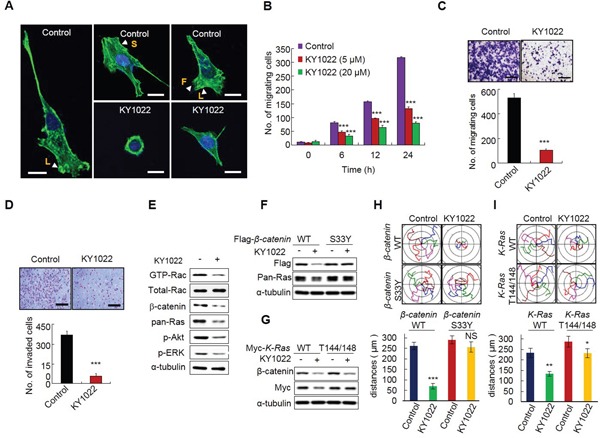
KY1022 inhibits actin rearrangement and suppresses the motility and invasion capabilities of CRC cells harboring *APC* and *K-Ras* mutations **A.** LoVo cells were grown and treated with either DMSO or KY1022 (20 μM), and cells were stained with phalloidin (green) and counterstained with DAPI (blue). Arrows indicate with L: lamellipodia, S: stress fiber, F: filopodia. Scale bars= 20 μm. **B.** Cells were grown in collagen coated dishes, and confluent cells were scratched and cell migratory behavior was monitored using real-time imaging. EGF (20 ng/mL) were used as chemoattractant. Data represent mean ± s.d. (n=3). **C.** LoVo cells treated with DMSO or KY1022 (20 μM) were subjected to Transwell migration assays. EGF (20 ng/mL) was used as chemoattractant. Data represent mean ± s.d. (n=3). **D.** Effect of KY1022 on the invasion assay using Matrigel-coated chambers. LoVo cells were seeded on matrigel-coated chambers. DMSO or KY1022 treated cells that invaded through the matrigel chambers were stained with 0.25% crystal violet. Representative images were captured (upper), the cells were counted from three independent experiments (bottom). Scale bars, 250 μm. EGF (20 ng/mL) was treated as a chemoattractant. Data represent mean ± s.d. (n=3). **E.** Immunoblot analyses of WCLs of LoVo cells treated with or without KY1022 (20 μM) using indicated antibodies. For GTP-Rac measurement, cell lysates were incubated with GST-PAK-CD, pulled-down, and analyzed by immunoblotting with an anti-Rac1 antibody. **F.-G.** Immunoblot analyses using WCLs of DLD1 cells stably expressing WT β-catenin, S33Y β-catenin, WT K-Ras, or T144/148A K-Ras treated with KY1022 (20 μM) with the indicated antibodies. **H.-I.** Effect of KY1022 on single cell migration ability was evaluated using DLD1 cells stably expressing WT β-catenin, S33Y β-catenin, WT K-Ras, or T144/148A K-Ras by real-time imaging. Migration distances were quantified. Experiments were performed five independent times. Data represent the mean ± s.d. (n=5). *P < 0.05, **P < 0.005, ***P < 0.001 by two sided Student's *t* test was used to determine statistical significance.

Since EMT is involved in actin rearrangement, we tested whether KY1022 inhibits the actin rearrangement of migratory LoVo colon adenocarcinoma cells derived from metastatic tumor nodules. The migratory LoVo cells formed stress fibers with the formation of filopodia beyond the leading edge of lamellipodia. However, actin bundles were concentrated in the peri-nucleus and cytoplasm with no stress fibers and filopodia formed around the cell periphery in cells treated with KY1022 (Figure 6A). The wound healing capacity of LoVo cells was reduced by KY1022 in a dose-dependent manner, as measured by real time imaging (Figure 6B). The inhibitory effects of KY1022 on the migration and invasion properties of LoVo cells were confirmed by the chamber migration and matrigel invasion assays, respectively (Figure 6C and 6D). Inhibition of cell migration and invasion by KY1022 may occur via inhibition of both the Ras-ERK and Ras-Akt pathways [30–32]. To clarify whether the inhibition effect of KY1022 on cell migration depends on the destabilization of β-catenin and Ras, we used 4 different DLD-1 cell lines stably expressing either WT β-catenin or WT K-Ras, and S33Y β-catenin or T144/148A K-Ras which abolish the degradation of β-catenin and Ras due to the mutations at their GSK3β-mediated phosphorylation sites [7]. The motilities of DLD1 cells expressing degradable β-catenin and K-Ras were efficiently inhibited by KY1022 (Figure 6F–6I), but this inhibitory effect was abolished in DLD1 cells stably overexpressing non-degradable S33Y β-catenin regardless of *K-Ras* mutational status (Figure 6F and 6H). DLD-1 cells overexpressing non-degradable K-Ras (T144/148A), in which WT β-catenin is degraded by KY1022 (Figure 6G), showed only a partial inhibitory effect on cell migration by KY1022 (Figure 6I). Therefore, the inhibitory effect of KY1022 on cell migration depends on the degradation of both β-catenin and Ras.

### KY1022 suppresses the EMT in small intestinal tumors of *APC^Min/+^*/*K-Ras^G12D^LA2* mice

**Figure 7:**
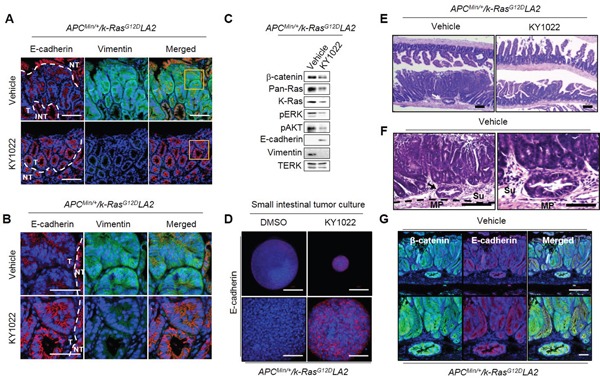
Effects of KY1022 on intra-tumor EMT in the small intestine of *Apc^Min/+^/K-Ras^*G12D*^*LA2 mice Five-week old male C57BL/6J-*Apc*^*Min*/+^*/K-Ras*^*G12D*^*LA2*mice (N=6) were injected i.p. with either KY1022 (25 mg/kg) or vehicle every day for 8 weeks **A.-B.** Formalin-fixed 4 μm paraffin sections of the mouse small intestine treated with either vehicle or KY1022 (25 mg/kg) were evaluated by IHC using by E-cadherin (red) and Vimentin (green) antibodies. Nuclei were counterstained with DAPI. Scale bar = 100 μm (A), Bottom scale bar = 50 μm (B), respectively. Images were captured using confocal microscopy (LSM700, Carl zeiss). T, tumor area, NT, Non-tumor area. **C.** Immunoblot analyses using WCL of small intestinal tumors of from *Apc*^*Min*/+^*/K-Ras*^*G12D*^*LA2* mice treated with either vehicle or KY1022 (25 mg/kg) Immunoblotting was were performed using the indicated antibodies. **D.** Small intestinal tumor cells of *Apc*^*Min*/+^*/K-Ras*^*G12D*^*LA2* were grown in matrigel 3D culture and E-cadherin expression was evaluated by ICC using tumor cells treated with either DMSO or KY1022 (20 μM) treated tumor cells. Nuclei were counterstained DAPI. The scale bars represent 200 μm (upper panel) and 50 μm (bottom panel), respectively. Representative images were captured using a confocal microscopy (LSM 700, Carl zeiss) confocal microscope. **E.** H&E images of vehicle or KY1022 treated small intestinal tumors of 12-week-old *Apc*^*Min*/+^*/K-Ras*^*G12D*^*LA2* mice were captured by microscopy (SMZ 745T, Nikon). Arrow indicates tumor invasion into submucosa. Scale bar = 1mm. **F.** H&E images of vehicle treated invasive tumors from *Apc*^*Min*/+^*/K-Ras*^*G12D*^*LA2* mice. Scale bar represents 100 μm (left) and 50 μm (right). MP, muscularis propria, Se, serosa. **G.** Four-micrometer tissue sections from invasive tumors of *Apc*^*Min*/+^*/K-Ras*^*G12D*^*LA2* mice were analyzed by IHC using the β-catenin and E-cadherin antibodies. Nuclei were counterstained with DAPI.

The enhancement of invasive properties in small intestinal tumors with a decreased E-cadherin expression was reported in mice with *APC* and *K-Ras* mutations [6]. We also observed invasions of small intestinal tumors with a significant reduction in E-cadherin expression in *APC*^*Min*/+^/*K-Ras*^*G12D*^*LA2* mice. In small intestinal tumors of *APC*^*Min*/+^ mice, E-cadherin was only mildly reduced, and invasions were not observed in *APC*^*Min*/+^ mice (Supplementary Figure S6A and S6B). However, intraperitoneal injection of KY1022 significantly increased and decreased the expressions of E-cadherin and vimentin, respectively, in *APC*^*Min*/+^*/K-Ras*^*G12D*^*LA2* mice (Figure 7A and 7B). Immunoblotting analysis also showed effective inhibition of the EMT phenotype by KY1022, with decreased levels of β-catenin, pan-Ras, K-Ras, p-ERK, and p-AKT in the small intestinal tumors of *APC*^*Min*/+^*/K-Ras*^*G12D*^*LA2* mice (Figure 7C). The increase in E-cadherin by KY1022 was also confirmed by using tumor cells derived from small intestinal tumors of *APC*^*Min*/+^*/K-Ras*^*G12D*^*LA2* mice (Figure 7D). Since the EMT phenomenon is associated with increased tumor invasiveness [6], we further investigated the effect of KY1022 on tumor invasion in *APC*^*Min*/+^*/K-Ras*^*G12D*^*LA2* mice. Tumor invasions, which occurred in *APC*^*Min*/+^*/K-Ras*^**G12D**^*LA2* mice were not observed in KY1022 injected *APC*^*Min*/+^*/K-Ras*^*G12D*^*LA2* mice (Figure 7E), whereas tumors of vehicle injected *APC*^*Min*/+^*/K-Ras*^*G12D*^*LA2* mice invaded to the submucosa and musculoris propria regions (Supplementary Figure S6B; Figure 7F). In addition, the epithelial origin of invasive tumors in vehicle injected *APC*^*Min*/+^*/K-Ras*^*G12D*^*LA2* mice was confirmed, as they stained tumor marker β-catenin and the epithelial cell marker E-cadherin (Figure 7G). Taken together, KY1022 effectively inhibited *APC* and *K-Ras* mutation driven intra-tumor EMT and following tumor invasion in *APC*^*Min*/+^/*K-Ras*^*G12D*^*LA2* mice.

## DISCUSSION

Aberrant activations of the Wnt/β-catenin and Ras signaling pathways play pivotal roles in EMT which triggers dissemination and budding of CRC cells, leading the primary tumor to distant sites such as the liver and lung. EMT is proposed as a critical mechanism for the acquisition of the malignant phenotype and confers motility and invasiveness to CRC cells during the initial stage of metastasis [33, 34], indicating that inhibition of EMT could be an ideal strategy for the suppression of progression and metastasis of CRC. EMT is histopathologically represented by the presence of tumor budding which retains aberrant Wnt/β-catenin signaling [19, 21, 22]. We previously observed that both Ras and β-catenin were increased in primary tumors compared with normal mucosa [7, 18]. Here, we observed that the levels of these two proteins were highly enriched in tumor buddings and were even higher than those observed in primary tumors. Recent studies have shown that small hair pin RNA-mediated knock down of mutant K-Ras significantly suppressed cell motility and invasiveness with increased E-cadherin expression in several cancer types including CRC [35–37]. In addition, selective degradation of specific oncogenic proteins has been indicated as an approach for anti-cancer therapy [15]. Therefore, reduction of β-catenin and Ras levels which are increased in CRC especially at tumor buddings could be an effective approach to inhibit metastatic CRC.

In this study, we identified KY1022 as a compound that most effectively inhibited the migration of LoVo cells among the compounds degrading both β-catenin and Ras via targeting Wnt/β-catenin pathway [18]. KY1022 significantly inhibited EMT phenotype both *in vitro* and *in vivo* resulting in the suppression of tumor invasions in small intestinal tumors of *APC*^*Min*/+^/*K-Ras*^*G12D*^*LA2* mice. The inhibitory effects of KY1022 on the migration and invasion of CRC cells harboring *APC* and *K-Ras* mutation correlated with its ability to inhibit the formation of stress fibers and the localization of lamellipodia and filopodia at the leading edge of migratory LoVo cells. KY1022 decreased the cell motility via destabilization of both β-catenin and Ras as shown by experiments with DLD-1 cells stably expressing non-degradable K-Ras mutant (Thr-144 and -148) or β-catenin mutant (S33Y). These results indicate that KY1022 effectively suppressed *APC* and *K-Ras* mutation-mediated EMT of CRC cells.

Overall, we identified and characterized a small molecule inhibiting the initiation and progression of mCRC. We specifically characterized the inhibitory effects of KY1022 at the early stage development of mCRC by suppressing the motility and invasiveness of cancer cells. Based on our recent identification that *APC* and *K-Ras* mutations synergistically enhance liver metastasis of CRC cells via stabilization of both β-catenin and Ras [9], small molecules destabilizing both β-catenin and Ras could be an effective therapeutic strategy to prevent metastasis to secondary organ. The apoptosis inducing effect of KY1022 on CRC would provide an added benefit in treating CRC. Further investigation of the inhibitory effects of KY1022 or its functionally improved analogs on metastases in association with cancer stem cell activations could lead to development of new drugs treating metastatic cancers and recurrences driven by aberrant activations of Wnt and Ras pathways.

## MATERIALS AND METHODS

### Cell culture, transfection and drug treatment

HEK293 cells, human CRC cells (LoVo, HCT15, and SW480), and CCD18-Co cells were obtained from the American Type Culture Collection (ATCC, Manassas, VA). Isogenic human DLD-1 CRC cell lines expressing either WT or MT *K-Ras* (D-WT and D-MT cells, respectively) were provided by B. Vogelstein (Johns Hopkins OncologyCenter) [29]. HEK293 reporter cell harboring the chromosomally incorporated TOPflash gene was provided by Dr. S. Oh (Kuk Min University, Seoul, Korea). HEK293 cells, HEK293 reporter cells and LoVo cells were cultured in DMEM containing 10% FBS. CCD18-Co cells were grown in DMEM supplemented with 20% FBS. SW480, HCT15, DLD1 cells were maintained in RPMI1640 medium (Gibco). For EMT experiments, D-WT and D-MT cells were maintained in DMEM containing 10% FBS and EGF (20 ng/mL). L-CM and Wnt3a-CM were prepared as previously described [38]. Lipofectamine2000 (Invitrogen) was used for plasmid transfection, according to the manufacturer's instructions. ALLN (25 μg/mL sigma-Aldrich), MG132 (20 μM; AMRESCO) were added to media to inhibit protein degradation. All chemicals were dissolved in dimethyl sulfoxide (DMSO; Sigma-Aldrich) for the *in vitro* studies.

### Immunoblotting

Cells were washed with ice-cold PBS and lysed with radio immunoprecipitation assay (RIPA) buffer [150 mM NaCl, 10 mM Tris pH 7.2, 0.1% sodium dodecyl sulfate, 1% Triton X-100, 1 % sodium deoxycholate, 5 mM ethylenediaminetetraacetic acid (EDTA)]. Proteins (10-20 μg) were separated using an 10-12% sodium dodecyl sulfate (SDS) polyacrylamide gel and transferred to a nitrocellulose membrane (Whatman). After blocking with 5% skim milk in tris buffered saline (TBS) containing 0.1% Tween20 (Sigma) for 2 hours, the membrane was incubated with the following primary antibodies: anti-β-catenin (BD bioscience), anti-pan-Ras monoclonal (Millipore), anti-K-Ras (Santa Cruz Biotechnology), anti-c-Myc (Santa Cruz Biotechnology), anti-APC (santa Cruz Biotechnology), anti-axin (Cell signaling Technology), anti-GSK3β (Cell signaling), Anti-p-GSK3β (Y216; BD Bioscience) anti-β-TrCP (Cell Signaling Technology), anti-p-β-catenin (S33/37/T41; cell signaling Technology), anti-p-ATF-2 (Cell Signaling Technology), anti-p-ERK (Cell Signaling Technology), anti-p-Akt (Cell Signaling Technology), anti-PCNA (Santa Cruz Biotechnology), anti-cleaved caspase3 (Cell Signaling Technology), anti-PARP (Santa Cruz Biotechnology), anti-α-tubulin (Cell Signaling Technology), anti-Flag (Cell Signaling Technology), anti-Myc (Santa Cruz Biotechnology), anti-GFP (Santa Cruz Biotechnology), anti-HA (Cell Signaling technology), anti-V5 (MBL International). Anti-p-Ras antibody (1:1,000) which recognizes phosphorylation of Ras proteins at T144 and T148 were described in our previous study [7]. Horseradish peroxidase-conjugated anti-mouse (Cell Signaling Technology) or anti-rabbit (Bio-Rad) antibodies were used as secondary antibodies. Five different CRC cell lines or 3 biological replicates of a single cell line were analyzed.

### Immunoprecipitation and ubiquitination assays

Immunoprecipitation and ubiquitination assays were performed as previously described [17]. Briefly, cells were washed in ice-cold PBS (Gibco) and lysed with RIPA buffer. the lysates were incubated with pan-Ras and protein G agarose beads (Thermo Scientific) or β-catenin and protein A agarose beads (Thermo Scientific) at 4°C for 12 hours, and the beads were then washed 3 times in RIPA buffer. The resulting immune complexes were resolved by SDS-PAGE, and immunoblotting was performed with the indicated antibodies. 3 biological replicates were performed.

### Reverse transcription and quantitative real-time PCR

HEK293 cells were seeded at a density of 3 x 10^5^ cells/well in 6-well plates and then treated with KY1022 (20 μM) with Wnt3a-CM for 24 hours. The cells were washed with ice-cold-PBS and total RNAs were isolated by using Trizol reagent (Invitrogen) as following manufacturer's instruction. Total RNA (2 μg) was reverse transcribed using 200U of reverse transcriptase (Invitrogen) in a 20 μL reaction carried out at 42°C for 1 hours. The following primer sets were used: *CTNNB1* (which encodes β-catenin), forward 5′-ACAAGCCACAAGATTACAAGAA-3′ reverse 5’-GCACCAATATCAAGTCCAAGA-3’; KRAS, forward 5’-AAACAGGCTCAGGACTTAG-3’ and reverse 5’-GTATAGAAGGCATCATCAAC-3’; GAPDH, forward 5’-CTGGTAAAGTGGATATTGTTG-3’ and reverse 5’-TGGAAGATGTGATGGGATTT-3’. 3 biological replicates were performed.

### MTT and colony formation assays

CRC cell lines (SW480, HCT15 and LoVo) were plated at a density of 7 × 10^3^ cells in 96 well plate, and HEK293 cells were seeded at a density of 1 × 10^4^ cells per 96 well plate. The cells were then treated with dimethyl sulfoxide (DMSO) or KY1022 for 24, 48 and 72 hours. 3-(4 5-Dimethylthiazol-2-yl)-2, 5-diphenyltetrazolium bromide (MTT; AMRESCO) was added to each well at a concentration of 0.25 mg/mL. After incubation for 2 hours at 37°C, insoluble purple formazan was obtained by removing the media and incubated in 100 μL DMSO for 1 hour. The absorbance of the formazan product was measured by FLUOstar OPTIMA (500 nm). For the colony formation assay, LoVo cells were seeded at a density of 500 cells per well in 12-well plates. The cells were treated with DMSO or KY1022 for 14 days. Media containing 5% serum was changed every 3 days. At the end of the experiment, cells were fixed in 10% neutral formaldehyde (Sigma) for 30 minutes in room temperature and stained with 0.5% crystal violet in 20% ethanol for 30 minutes. Mean ± s.d. are reported, based on 3 biological replicates.

### Flow cytometry analysis

CRC cell lines (D-WT, D-MT and SW480 cells) were plated at a density of 2 × 10^4^ onto 6-well plates and treated with 20 μM KY1022 or DMSO for 36 hours. The cells were harvested and resuspended in binding buffer (10 mM HEPES, 140 mM NaCl, and 2.5 mM CaCl_2_) at 1 × 10^6^ cells/mL. 200 μL of cells in Annexin ν binding buffer were incubated with 5 μL annexin ν -FITC (Sigma-Aldrich) and 10 μL PI (Sigma-Aldrich) for 15 minutes in the dark. A volume of 800 μL of Annexin ν Binding buffer were added to each tube, and analyzed by Flow cytometry (BD bioscience). Mean ± s.d. are reported, based on 3 biological replicates.

### Wound healing assay

LoVo cells were coated with collagen (500 μg/mL) were coated in 6-well or chamber. After grown confluent, cells were scratched with 200 μL tip. And then change the 10% DMEM containing EGF (20 ng/mL) with or without KY1022 (20 μM). Wound healing was recorded using a time-lapse video microscope (Eclipse *Ti*, Nikon) in a humidified 5% CO_2_ incubator at 37°C. The number of cells that migrated to heal the wound was quantified using the NIS-Elements AR 3.1 software (Nikon). Screenshots were captured from video files and were represented as images at several time points. Mean ± s.d. are reported, based on 3 or 5 biological replicates.

### Time-lapse microscopy

LoVo, D-WT and D-MT cells were seeded at a density of 1 x 10^4^ on collagen (500 μg/mL) coated chamber. After 24 hours, change the media containing EGF (20 ng/mL) with or without KY1022 (20 μM). Single cells were imaged using a time-lapse video microscope (Eclipse *ti*, Nikon). The videos and the migratory paths of single cells were constructed using NIS-Elements AR 3.1 (Nikon). Screenshots were captured from the movie file and represented as images. Mean ± s.d. are reported, based on 5 biological replicates.

### Invasion assay

EGF (20 ng/mL) treated LoVo cells were seeded at a density of 1 x 10^5^ on matrigel coated chamber (BD bioscience) with or without treatment with KY1022 (20 μM). Cells were allowed to invade for 18 hours. After clearing the cells on the inner surface of the chamber, the cells on the outer surface were fixed using 10% neutral formaldehyde (Sigma) for 15 minutes and stained with crystal violet for 15 minutes. The chambers were dipped in distilled water to remove the excess staining and allowed to dry. Representative images were captured by microscope (TE-2000U, Nikon). Mean ± s.d. are reported, based on 3 biological replicates. Mean ± s.d. are reported, based on 3 or 5 biological replicates.

### Rac activation assay

LoVo cells were seeded at a density of 2 x 10^6^ in 100 mm dishes. DMSO or KY1022 (20 μM) were treated for 18 hours. After harvest, lysates of DMSO or KY1022 treated LoVo cells were incubated with bacterially produced GST-PAK-CD fusion protein bound to glutathione-coupled agarose beads. The proteins bound to the fusion protein were washed three times with lysis buffer, eluted in 3× Laemmli sample buffer, and analyzed for GTP-Rac by immunoblotting with an anti-Rac1 antibody.

### Immunocytochemistry and phalloidin staining

LoVo, D-WT and D-MT cells were grown in collagen coated (500 μg/mL) cover slip. Cells were treated DMSO or KY1022 (20 μM) with EGF (20 ng/mL) for 18 hours. Cover slips were fixed with 10% neutral formaldehyde for 2 hours, followed by permeabilization with 0.1% Triton X-100 for 30 minutes, blocking in 5% bovine serum albumin (BSA) for 1 hours. The cover slips were incubated with primary antibody overnight at 4°C, and followed by with anti-mouse Alexa Flour 488 (Life Technologies) or anti-rabbit Alex Flour 555 (Life Technologies) secondary antibody (1:500) for 1 hour at room temperature. Primary and secondary antibodies were diluted with PBS containing 1% BSA and 1% normal goat serum (NGS; Vector Laboratories). The cover slips were then counterstained with 4', 6'-diamidino-2-phenylindole (DAPI; Sigma-Aldrich) and mounted in Gel/Mount media (Biomeda Corporation). For cytoskeleton staining, cells were fixed with 10% neutral formaldehyde, permeabilized, blocked, and incubated with Alexa Fluor 568 phalloidin (MolecularProbes) for 30 minutes. Immunofluorescent images were captured using confocal microscopy (LSM 700, Carl Zeiss). At least 3 fields per section were analyzed.

### Animal model and analysis of tumor tissue

C57BL/6J-*Apc^Min/+^*(*Apc^Min/+^*) and B6.129S-*Kras^tm3Tyj^* (*K-Ras^G12D^LA2*) mice were obtained from Jackson Laboratory (Bar Harbor, ME) or the National Cancer Institute mouse repository (National Institutes of Health Technology Transfer Center, Bethesda, MD). To generate *APC*^*Min*/+^*/K-Ras^G12D^LA2* compound mice, *Apc*^*Min*/+^ mice were crossed with *K-Ras*^*G12D*^*LA2* mice. Mouse genotyping was performed using genomic DNA extracted from the tail. To control for genetic background effects, sex-matched littermates were always used as controls. To investigate the *in vivo* efficacy of KY1022, *APC*^*Min*/+^/*K-Ras*^*G12D*^*LA2* (*n* = 6) mice were injected i.p. with KY1022 (25 mg/kg) every day for 7 weeks. Immediately after sacrifice, the abdomen of each mouse was cut open longitudinally and cleaned by flushing with cold PBS for 2 times. After washing vigorously, resected tissues were fixed with 10% neutral formaldehyde (Sigma) for 24-48 hours in 4°C. Fixed tissues were embedded in paraffin according to standard procedures. The tumors were classified according to standard World Health Organization histopathological criteria. Small, medium and large size of tumors were classified by diameter under 1 mm, between 1 mm and 3 mm, over 3 mm, respectively. For immunoblotting analyses, a subset of freshly isolated tissues were snap frozen in liquid nitrogen and stored at −80°C. Mean ± s.d. are reported, based on 6 mice for each group.

### Immunohistochemistry

For immunohistochemical analysis, 4 μm paraffin embedded tissue sections were deparrafinized and rehydrated and autoclaved with 10 mM citrate buffer (pH 6.0) for 15 minutes. The sections were then blocked with 5% BSA and 1% NGS in PBS for 30-60 minutes. After blocking, sections were incubated with a primary antibody; anti-β-catenin (BD bioscience), anti-pan-Ras monoclonal (Millipore), anti-cleaved caspase3 (Cell Signaling Technology), anti-cleaved PARP (Cell Signaling Technology), or anti-PCNA (Santa Cruz Biotechnology) overnight at 4°C, followed by secondary antibodies (1:500) for 1 hour at room temperature. Primary and secondary antibodies were diluted with PBS containing 1% BSA and 1% NGS. The sections were then counterstained with DAPI (Sigma-Aldrich) and mounted in Gel/Mount media (Biomeda Corporation). All incubations were conducted in dark, humid chambers. The fluorescence signal was visualized using confocal microscopy (LSM700, Carl Zeiss) at excitation wave lengths of 488 nm (Alexa Fluor 488), 543 nm (Alexa Fluor 555), or 405 nm (DAPI). At least 3 fields per section were analyzed to establish statistical significance. For peroxidase IHC analysis, sections were blocked by 3.42% H_2_O_2_ and incubated with primary antibody overnight at 4°C, followed by incubation with biotinylated anti-mouse (Dako) or biotinylated anti-rabbit (Dako) secondary antibodies for 1 hour at room temperature. The samples were then incubated in ABC kit (Vector Laboratories) for 1 hour, stained with 3, 3′-diaminobenzidine (DAB; Dako) for 3−7 minutes and counterstained with Mayer's hematoxylin (Muto). All incubations were conducted in humid chambers. Signals were analyzed using a bright field microscope (Nikon ECLIPSE 80i). Expression of β-catenin or pan-Ras as indicated in IHC images was quantified using IHC profiler from images. At least 3 fields per section were analyzed.

### Primary tumor cell culture

In mouse tumor organoid culture, small intestinal tumors of *APC^Min/+^/K-Ras^G12D^LA2* were isolated and flushed with ice cold PBS for 3 times and tumors were isolated to single cell by 0.25% trypsin for 30 minutes, and then mix with ice cold matrigel (300 cells/ 30 μL of matrigel) and cell/matrigel mixture were cultured in N2 media containing 50 ng/mL EGF, 50 ng/mL bFGF and 500 μg/mL R-spondin1 [39]. Medium was freshly changed every 2 days.

### TMA sample analysis

TMAs for normal and colon adenocarcinoma and metastatic adenocarcinoma (CO702) were purchased from US Biomax, and IHC analyses was performed with β-catenin or Ras as described in previous studies [40]. The TMA slides were visualized by microscopy (Eclipase 80i, Nikon). For quantitative analysis, H-Score of each staining was determined by IHC profiler software. H-Score = 3* highly positive population + 2 * Positive population + 1* weak positive population + 0* negative population.

### Statistical analyses

All statistical analyses were performed using Microsoft Excel spreadsheets or GraphPad Prism5 Software as described previously [18]. Group differences were determined with the Student *t* test. Data are expressed as means and standard deviation. All statistical tests were two-sided, and *P* values less than 0.05 were considered statistically significant.

## SUPPLEMENTARY MATERIALS FIGURES


