# Supplementary material for: KY1022, a small molecule destabilizing Ras via targeting the Wnt/β-catenin pathway, inhibits development of metastatic colorectal cancer
Source: Oncotarget. 2016 Nov 7;7(49):81727–40. doi: 10.18632/oncotarget.13172 (PMC5348425; doi:10.18632/oncotarget.13172)
Supplement: Supplementary file 1 [file oncotarget-07-81727-s001.pdf]

# KY1022, a small molecule destabilizing Ras via targeting the Wnt/β-catenin pathway, inhibits development of metastatic colorectal cancer

## SUPPLEMENTARY FIGURES

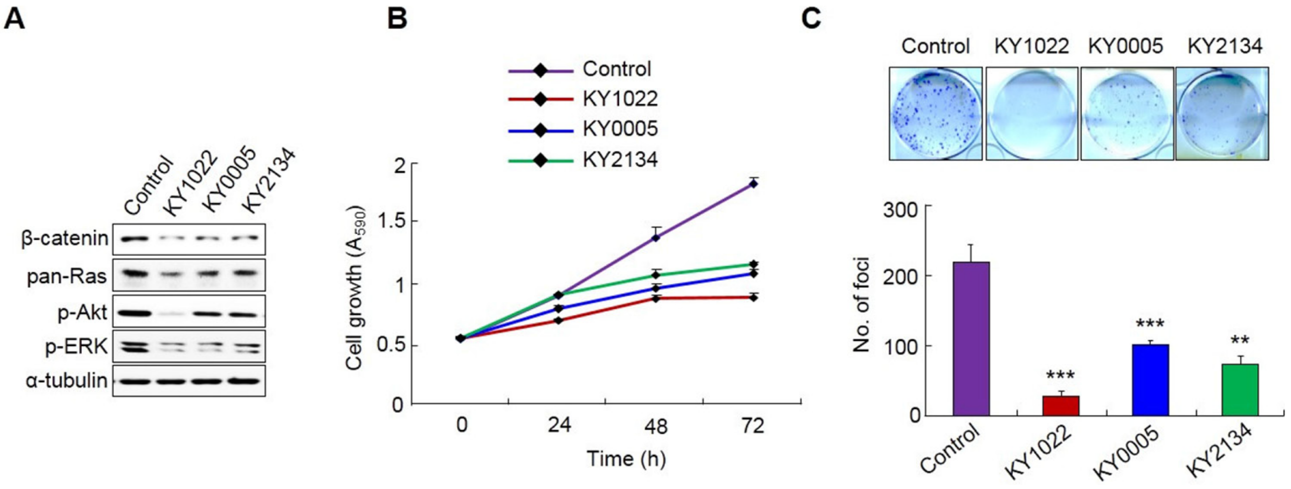


**Supplementary Figure S1: Effects of representative small molecules that degrades both β-catenin and Ras on the cell growth and transformation of LoVo CRC cells.** LoVo cells were treated with each compound at a concentration of 20 μmol/L. **A.** WCLs from LoVo cells treated with each chemicals were subjected to immunoblotting analysis using the indicated antibodies. Data represent mean ± s.d. (n=3). **B.** MTT assays to compare the effect of each compound on cell growth at indicated time points. **C.** Colony formation assays to compare the effect of each compound on colony forming ability. Data represent the mean ± s.d. (n=3). ***P* < 0.005, ****P*<0.001 by two sided student t test between control and each samples, respectively.

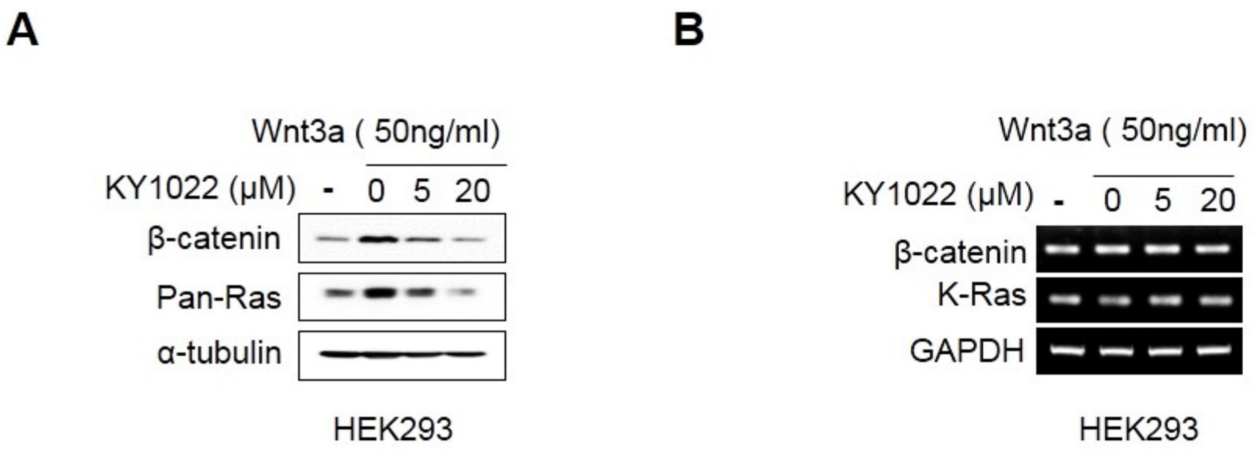


**Supplementary Figure S2: Effects of KY1022 on the stabilities of β-catenin and Ras.** HEK 293 cells grown in DMEM containing Wnt3a (50ng/mL) were treated with KY1022 (0, 5, or 20 μM) for 24 hours **A.** WCLs from KY1022 treated HEK293 cells were immunoblotted using the indicated antibodies. **B.** Cells treated with KY1022 were subjected to quantitative real-time PCR.

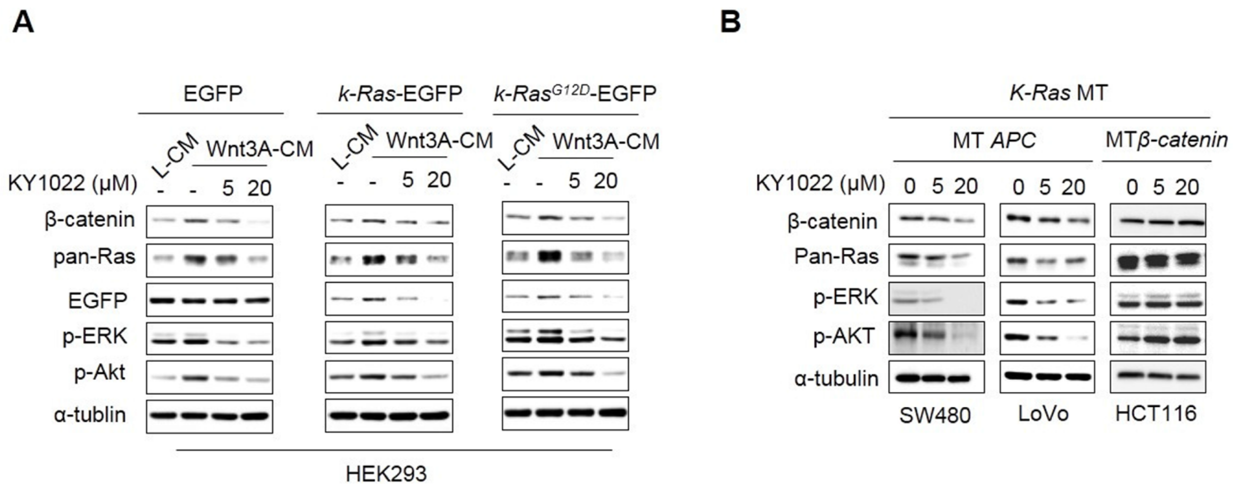


**Supplementary Figure S3: Effects of KY1022 on the stabilities of β-catenin and Ras, and their downstream kinase activities. A.** HEK293 cells were transfected with either pCDNA3.1-EGFP or plasmid harboring *K-RAS*$^{WT}$ or *K-Ras*$^{G12D}$ with the EGFP tag, and KY1022 (0, 5, 20 μM) along with Wnt3a-CM in each cells. WCLs from each cells and treatment type were subjected to immunoblotting analysis. **B.** Immunoblot analyses using WCLs of KY1022-treated cells (0, 5, 20 μM). (A-B). Immunoblotting were performed using the indicated antibodies.

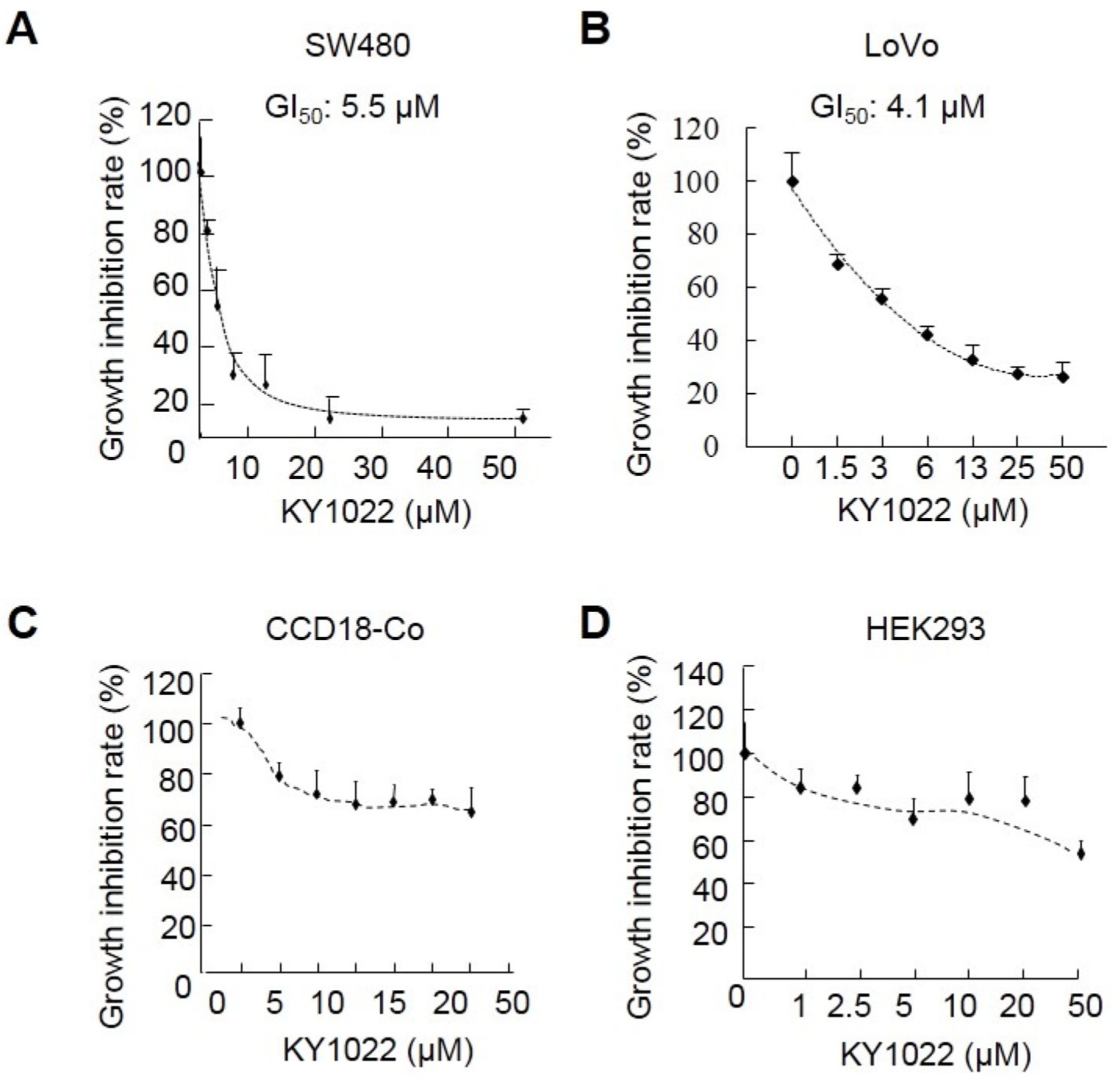


**Supplementary Figure S4: Effects of KY1022 on the growth of CRC cells and normal cells.** SW480 **A.** LoVo **B.** CCD18-Co **C.** and HEK293 **D.** cells were treated with various concentrations of KY1022 for 4 days. Cell growth was quantified using an MTT assay and normalized to DMSO-treated control. Data represent the mean ± s.d. (n=3).

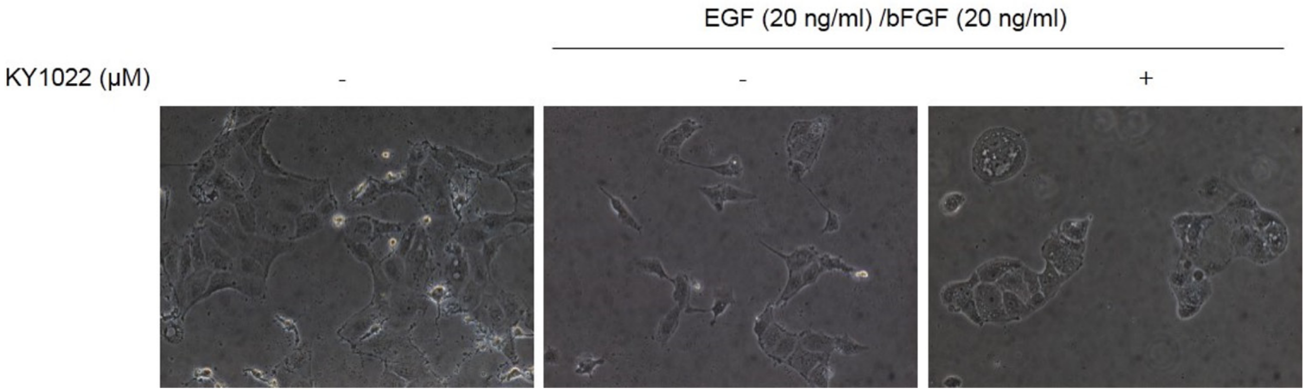


**Supplementary Figure S5: Effect of KY1022 on EGF- and bFGF-mediated morphological changes in MDCK cells.** MDCK cells were treated with KY1022 (20 μmol/L) for 14 days in DMEM supplemented with 10% FBS with or without EGF (20 ng/mL) and bFGF (20 ng/mL). Microscope images were captured using a microscopy (TE2000U, Nikon).

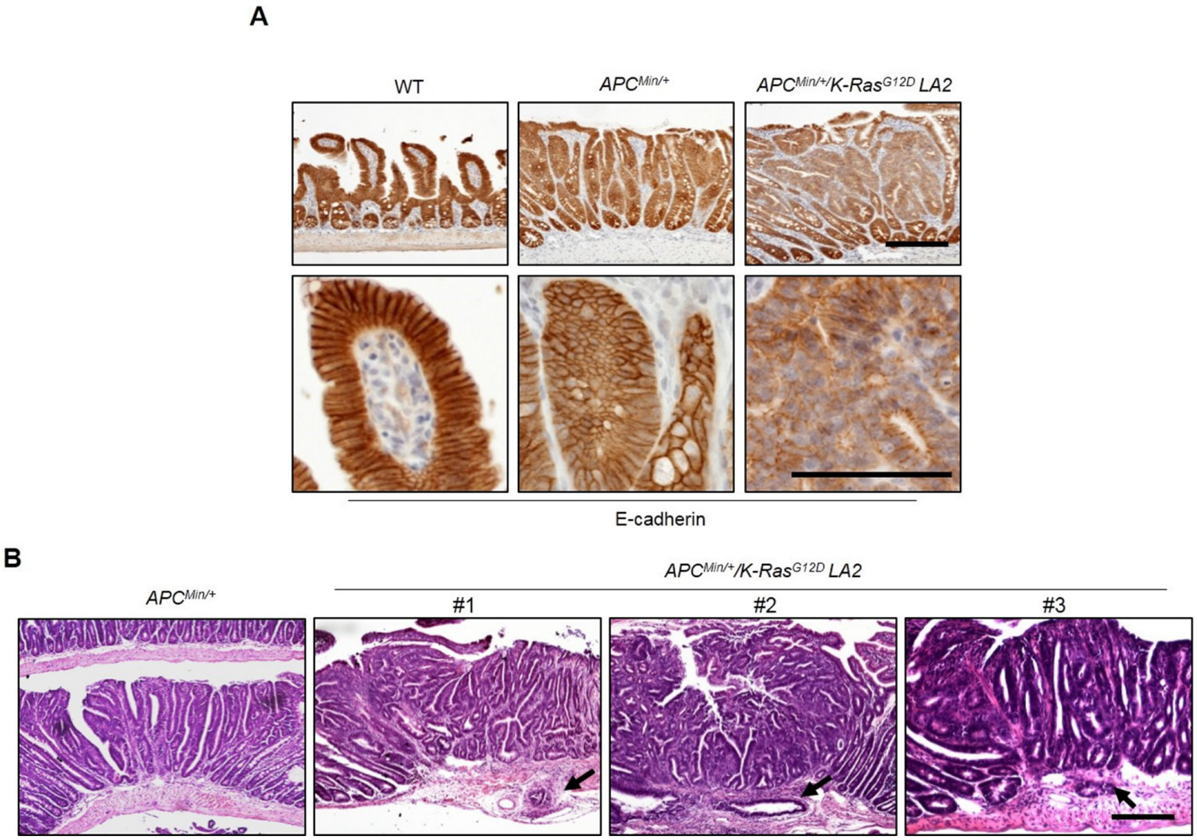


**Supplementary Figure S6: Effects of oncogenic *K-Ras*$^{G12D}$ on the loss of E-cadherin and Tumor invasion in small intestinal tumors in *APC*$^{Min/+}$/*K-Ras*$^{G12D}$LA2 mice.** Paraffin embedded formalin-fixed 4 µm paraffin sections of small intestinal tumors of 12-week old WT, *APC*$^{Min/+}$ and *APC*$^{Min/+}$/*K-Ras*$^{G12D}$*LA2* mice were evaluated with IHC and H&E analyses. Representative images of DAB **A.** and H&E staining **B.** respectively. Arrows represent invaded tumors. Scale bar = 100 µm.
